# Meeting of the Erie County Medical Society on Account of the Death of Dr. Treat

**Published:** 1861-09

**Authors:** 


### MEETING OF THE ERIE COUNTY MEDICAL SOCIETY ON ACCOUNT OF THE DEATH OF DR. TREAT.

The Society was called to order by Dr. Eastman, President, in the Chair. In the absence of the Secretary, Dr. Gay was appointed *pro tem.*

The President announced, in a few feeling and appropriate remarks, the death of Dr. William Treat, and invited the Society to take such action as was deemed proper. Dr. Treat had been a member since 1844.

Dr. Strong moved a committee be appointed to draft resolutions expressive of the sense of the Society.

Dr. White said it was with no ordinary emotions he arose to second the motion of Dr. Strong. There was no more gentle or modest man in the profession than Dr. Treat. He was always a student, and practiced medicine as a scientific avocation. Had he possessed the means to have raised himself above the drudgery of the profession, he would have been a philosopher. He had a mind for natural history. His motto, of which he made frequent use, was characteristic of the man, viz: "To the pure all things are pure." He was self-abnegating. He visited a poor patient while laboring under the disease of which he died. He was exemplary in all his relations in life.

Dr. White said when Dr. Treat came to this city in 1844 he brought with him a letter of introduction from the father of Gen. McClellan, then the most brilliant surgeon of America; and from that time to the present, there had never existed any other than the most pleasant and friendly relations between them. Professional difficulties could never arise between Dr. Treat and any other Physician, from any action of his, for he was altogether above anything unprofessional.

Drs. Strong, White, and Samo were appointed a committee on resolutions. Dr. Strong, as Chairman, after consultation, reported the following:

Inasmuch as by a stroke of an All Wise Providence, at once sudden, inscrutable and afflictive, our professional brother Dr. Wm. Treat has been called away from our side and from his earthly labor, therefore, as a feeble expression of the sense of this Society,

*Resolved,* That in the death of Dr. Treat, our Society deplores the loss of one who was ever ready to discharge faithfully and fully his share of its duties and responsibilities—our profession, the loss of one who possessed in rare combination scholarly tastes, and purpose, with practical tact and skill, and a conscientious and self-forgetting devotion to the well-being of those entrusted to his care—and Society at large, the loss of one of its most guileless, unselfish and intelligent citizens.

*Resolved*, That great as is our grief at his departure, we feel it to be trivial compared to the irreparable loss of those near and dear to him in the sanctuary of home; and while we tenderly commend them to the God of the widow and the Father of the fatherless for true solace; we do hereby also tender to the stricken widow and family an expression of our sincere and profound sympathy, impotent to relieve though it be, in this their dark hour.

*Resolved*, That as a Society we will attend the funeral of our departed brother and friend, and that our President designate suitable names from the membership to act as pall-bearers, and that we wear the usual badge of mourning for thirty days.

*Resolved*, That a copy of the action of the Society be presented to the widow and family, and one also to the Press of the city, and to the Buffalo Medical Journal, for publication.

Dr. Strong remarked that the resolutions failed in expressing his own estimate of the deceased. He had known him for fifteen years, and had never known a more gentle, kind hearted man, or a more acute and dispassionate observer of men. His serene and cheerful temper, added to his remarkable attainments, made him one of the most delightful companions. He felt to mourn for his loss as a brother beloved.

Dr. C. C. F. Gay remarked that, with the deceased, he had, for a few years past sustained the most pleasant and intimate relationship. Modest and retiring, he never sought that professional advancement which belonged to him of right; and when the past year, although declining to stand as a candidate for the presidency of this society, when unanimously elected, he entered upon its duties with much pride and zeal, and left within its archieves two addresses, marked by an ability characteristic of their able author. His last address, the product of his superior intellect, as an exhibition of scientific attainment, a wonder and a marvel, will live long after the present generation of physicians have passed away.

Dr. William Treat, the skillful physician, the kind and devoted husband and father, the generous friend, the consistent Christian, and that "noblest work of God," an honest man, has gone to enter upon his blessed reward. We, his fellow physicians, have this night met with hearts too full for utterance, to pay this, our last tribute of respect to the worth, and to mourn and deplore the loss, of our departed brother.

May God, from out his abundance, richly provide for the widow and orphan.

After Dr. White had given an informal account of the sickness of Dr. T., the resolutions were adopted and the meeting adjourned.

